# Dual-Functional Microcapsules with Antibacterial and Self-Healing Properties for Dental Resin

**DOI:** 10.3390/polym18212739

**Published:** 2026-11-04

**Authors:** Hongze Che, Minghui Zhang, Wei Wu, Jialiang Liu, He Wang, Qing Lu

**Affiliations:** 1 School of Dentistry, Beihua University, Jilin 132013, China; chehz@beihua.edu.cn (H.C.);; 2 School of Clinical Medicine, Beihua University, Jilin 132013, China; 3 VIP Integrated Department, Hospital of Stomatology, Jilin University, Changchun 130021, China

**Keywords:** microcapsules, quaternary ammonium methacrylate, antibacterial activity, dental resins, self-healing

## Abstract

Background: Current self-healing microcapsules for dental composites have been developed toward multifunctional properties. Antibacterial microcapsules are commonly loaded with antibacterial agents on their surfaces. Nevertheless, restricted microcapsule incorporation within composite resin limits their antibacterial properties. For the complex oral environment, further enhancement of antibacterial properties of microcapsules is still required; Methods: This study aimed to fabricate microcapsules co-encapsulating non-polymerized methacryloyloxyethyl trimethyl ammonium chloride (METAC) and healing monomers via in situ polymerization. The morphology, particle size distribution, chemical composition, thermal stability and antibacterial activity were characterized. The experimental dental resin containing the microcapsules was fabricated, and its flexural properties, antibacterial activity, self-healing efficiency and cytotoxicity were evaluated; Results: The 6 wt% METAC loading and 10 wt% microcapsule incorporation ratio were selected within the investigated ranges. At this formulation, the surface-scratched dental resin exhibited 61.82% antibacterial efficiency against *S. mutans* relative to the METAC-free control, with no significant decline in activity after 28-day aging. It also achieved a self-healing efficiency of 25.7% and showed no discernible cytotoxicity under the tested in vitro conditions; Conclusions: The prepared microcapsules achieved antibacterial and self-healing properties, and no statistically significant difference in self-healing gain was detected between the METAC-loaded and METAC-free groups. Incorporation of these microcapsules into dental resin is expected to enhance its antibacterial potential.

## 1. Introduction

Dental composite resins are commonly used as restorative materials for the repair of tooth defects. However, fracture and secondary caries that develop during long-term clinical service are the primary causes of restoration failure [1,2,3,4]. Endowing composite resins with self-healing properties has shown promise in the extension of their service life. Upon the formation of microcracks within the resin, the material repairs itself independently, thereby preventing the further propagation of cracks that may result in fracture. Wertzberger et al. first investigated the application of self-healing microcapsules in dental composite resins and confirmed their self-healing potential [5]. Subsequently, Wu used dental methacrylate monomers as the primary healing material along with a redox curing system [6]; it significantly enhanced the restorative effectiveness of composite resins and established the groundwork for the development of self-healing microcapsules.

Thereafter, researchers have focused on optimizing the performance of microcapsules. To balance the rigidity and elasticity of the urea–formaldehyde microcapsule wall, Rajasekaran used a combination of melamine and N,N-dimethylacrylamide [7]. Huynh used silane coupling agents to treat the microcapsule surfaces. Surface functionalization strengthened the interfacial link between microcapsules and the composite resin matrix; furthermore, it enhanced self-healing efficacy [8]. According to Khan, new-generation dental materials should not be restricted to a particular repair function. Rather, self-healing should be combined with multiple functional mechanisms to offer auxiliary features [9]. Zhang created new composite resins by combining expandable monomers with 7.5 wt% microcapsules. The resulting composite resins showed minimal polymerization shrinkage and satisfactory self-healing efficiency [10]. Taut created hydroxyapatite (HAP)-loaded self-healing microcapsules. HAP facilitated maintaining the structural integrity of the capsule shells and endowed the composite resins with the ability to remineralize [11]. Qiu synthesized aggregation-induced emission luminogen microcapsules. These microcapsules provide warning signals after the appearance of cracks in the composite resins [12].

The integration of antibacterial functions to reduce the incidence of secondary caries has emerged as a mainstream research area in the development of multifunctional dental composite resins. Dental products frequently utilize polymerizable quaternary ammonium monomers (QAMs) as antibacterial agents [13,14]. Each QAM molecule consists of polymerizable methacrylate groups that covalently attach to the resin network after curing; they exert bactericidal effects against cariogenic bacteria through a contact-killing mechanism [15,16]. QAMs do not always impair the overall performance of composite resins when used within a suitable dosage range. Wu incorporated dimethylaminohexadecyl methacrylate, amorphous calcium phosphate nanoparticles, and microcapsules into dentin adhesives. The modified adhesives concurrently offered long-term antibacterial properties, remineralization, and self-healing performance [17]. To give composite resin stable antibacterial qualities, other researchers have grafted antibacterial components onto inorganic fillers and placed these fillers on microcapsule surfaces [18,19]. However, the antibacterial efficacy of such systems is highly dependent on microcapsule dispersion uniformity. Meanwhile, microcapsule incorporation is commonly found to impair the mechanical properties of composite resins, and thus their loading is restricted, which ultimately results in limited antibacterial activity. In addition, resin damage may increase the risk of secondary caries at the restoration–tooth interface [20,21]. Therefore, more effective antibacterial strategies are urgently needed.

In this study, we hypothesized that encapsulating the antibacterial agent within the microcapsules would trigger the antibacterial activity at the site and time of damage, while retaining acceptable mechanical and biological properties. To achieve this, healing methacrylate monomers and non-polymerized QAMs were co-encapsulated within microcapsules at a specific ratio. The two essential functional components are simultaneously released upon microcrack rupture, allowing for crack repair and favorable antibacterial activity. Meanwhile, the released QAMs are expected not to affect the self-healing process. Intact microcapsule shells prevent core leakage, thus maintaining the antibacterial activity of the encapsulated QAMs. Additionally, this encapsulation strategy may reduce unnecessary long-term exposure of the oral environment to the antibacterial agent.

## 2. Materials and Methods

### 2.1. Materials

Urea (purity ≥ 99.5%), formaldehyde solution (37%), 2,2-bis [4-(2-hydroxy-3-methacryloyloxypropoxy)phenyl]propane (Bis-GMA), and triethylene glycol dimethacrylate (TEGDMA) were purchased from Aladdin (Shanghai, China). Methyl vinyl ether–maleic anhydride copolymer (EMA) was sourced from Sigma-Aldrich (St. Louis, MO, USA); 2-Hydroxyethyl methacrylate (HEMA), [2-(Methacryloyloxy)ethyl]trimethylammonium chloride (METAC), phenylbis(2,4,6-trimethylbenzoyl)phosphine oxide (BAPO), benzoyl peroxide (BPO), and N,N-dihydroxyethyl-p-toluidine (DHEPT) were obtained from Macklin (Shanghai, China). Artificial saliva was purchased from Yuanye Biotechnology (Shanghai, China). *Streptococcus mutans* (*S. mutans*, ATCC 25175) was purchased from BioBW Biotechnology (Beijing, China). The HaCaT human keratinocyte cell line was purchased from Meisen Cell (Hangzhou, China). The Cell Counting Kit-8 (CCK-8) was sourced from Proteintech (Wuhan, China), and the LIVE/DEAD Bacterial Staining Kit was purchased from Beyotime (Shanghai, China).

### 2.2. Synthesis of Microcapsules

**Figure 1:**
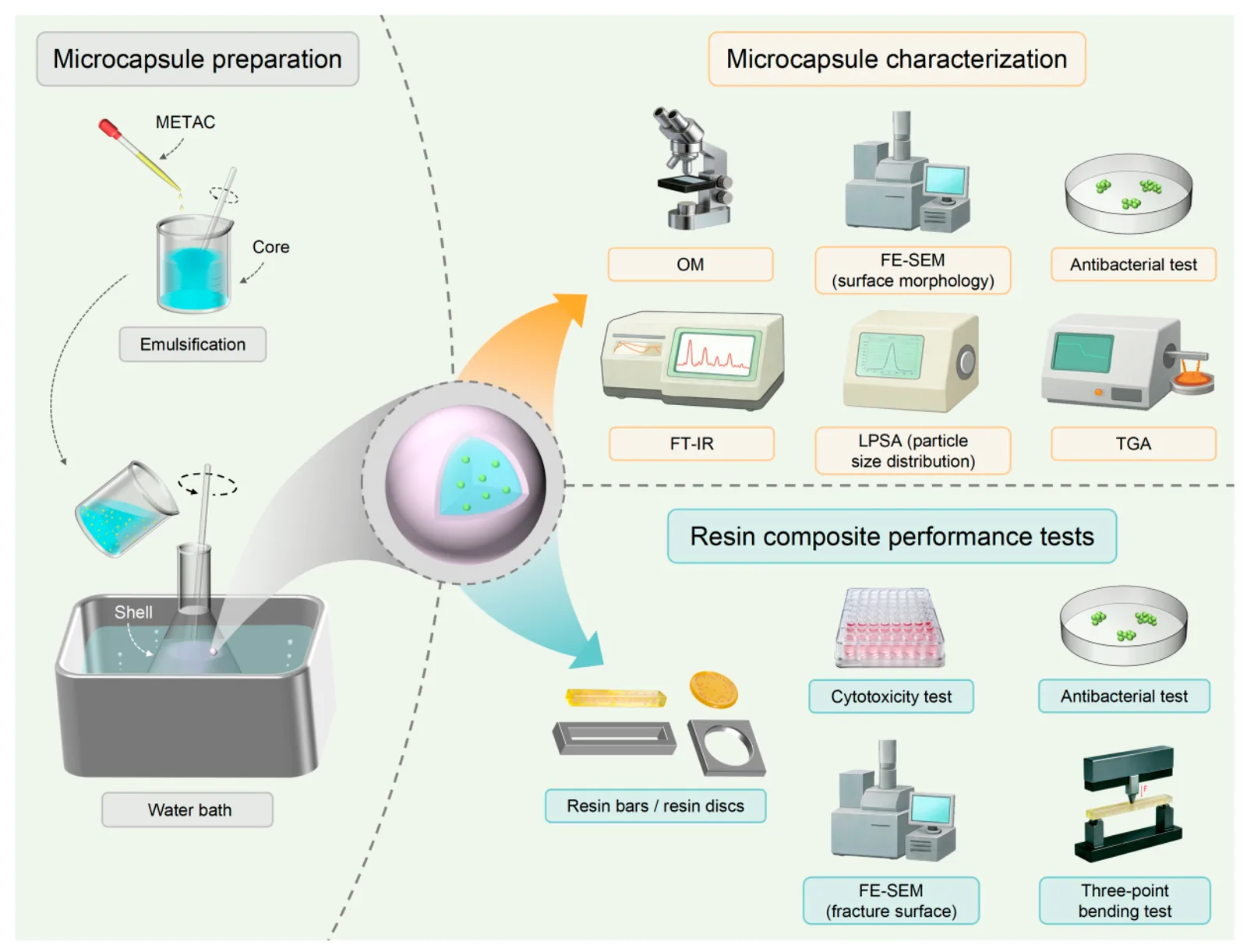
Schematic diagram of experimental procedures. Some graphic elements in this figure were generated using Jimeng (version 2.3.9, Shenzhen, China).

Figure 1 shows a schematic overview of the experimental procedure. In situ polymerization was used for microcapsule preparation. The core monomers were prepared at a 1:2 mass ratio of HEMA to TEGDMA, with a total mass of 3.5 g. METAC, a characteristic QAM, was used as the antibacterial agent. It was added dropwise at gradient loadings of 2 wt%, 4 wt%, 6 wt%, and 8 wt% relative to the total core monomer mass. DHEPT was then added at 1 wt% as an accelerator for the subsequent redox initiation system. All loadings reported in this study refer to the nominal amounts introduced during synthesis. The core mixture was continuously stirred until homogeneous. For emulsification, 10 mL of 4 wt% EMA aqueous solution was added to the core mixture. It was mechanically stirred at 500 rpm for 1.5 h at room temperature to obtain a stable oil-in-water (O/W) emulsion. Next, 0.82 g of urea and 1.92 g of formaldehyde solution were dissolved in 10 mL of deionized water, and the pH was adjusted to 8.5. To prepare a transparent urea–formaldehyde (UF) prepolymer solution, the mixture was reacted at 75 °C for 1.5 h with continuous stirring at 200 rpm. Under continuous stirring, the core emulsion was added dropwise into the UF prepolymer solution, and the system pH was maintained at 3.5. The reaction was allowed to proceed at 55 °C for 4 h under stirring at 200 rpm. Finally, the product was rinsed with deionized water and centrifuged at 160× *g* for 1 min over three cycles; the supernatant was discarded. Microcapsules were obtained by drying the resulting precipitate. Microcapsules with each METAC loading were synthesized in a single batch.

### 2.3. Microcapsule Characterization Methods

Microcapsules were characterized through a sequence of complementary steps. First, optical microscopy (OM, Olympus BX53, Tokyo, Japan) was used to visualize the in situ polymerization process and the microcapsule morphology. The surface morphology was further characterized via field emission scanning electron microscopy (FE-SEM, HITACHI SU8000, Tokyo, Japan). Subsequently, a laser particle size analysis (LPSA, Malvern Mastersizer 3000, Malvern, UK) was used to measure their particle-size distribution. The chemical composition was analyzed using a Fourier transform infrared spectrometer (FT-IR, Thermo Nicolet iS5, Waltham, MA, USA) via the attenuated total reflectance method. Finally, a thermogravimetric analysis (TGA, HITACHI STA200, Tokyo, Japan) up to 600 °C was used to assess the thermal stability of the microcapsules. The baseline-tangent intersection approach was used to calculate extrapolated onset decomposition temperatures, and the same criteria were used for each examined sample.

### 2.4. Antibacterial Activity and Stability of Microcapsules

*S. mutans* was resuscitated from frozen stocks and cultured anaerobically in a Brain Heart Infusion (BHI) broth at 37 °C. After establishing a 24-h growth curve, a 10-h mid-logarithmic suspension (OD_600_ = 0.78) was used for further experiments. First, a two-factor experimental design (*n* = 3 per group) was used to investigate the impact of METAC loading ratios (0–8 wt%), microcapsule integrity (intact or crushed). Then, the effects of microcapsule integrity and aging treatment (non-aged or aged in artificial saliva for 28 d) were evaluated. Aging was performed at 37 °C with the specimens immersed in 10 mL of artificial saliva, replaced every 3 days. Crushed microcapsules were obtained by mechanical grinding using glass rods. Each system consisted of 5 mL of BHI broth, 500 μL of bacterial suspension, and 500 μL of microcapsule suspension (20 mg/mL). The control group received 500 μL of phosphate-buffered saline (PBS) instead of the microcapsule suspension. After 24 h of co-culture, bacterial suspensions underwent 10-fold serial dilutions and were spread onto BHI agar plates; colony-forming units (CFU) were measured to calculate surviving bacteria (CFU/mL). Equation (1) was used to calculate the antibacterial efficiency. CFU counting and microscopy image analysis were performed by operators blinded to group allocation.

Antibacterial efficiency (%) = (CFU_control_ − CFU_experimental_)/CFU_control_ × 100% (1)

### 2.5. Preparation of Microcapsule-Loaded Resin Specimens

First, 1 wt% BPO (oxidizing agent of the BPO-DHEPT redox initiation system for self-healing upon microcapsule rupture) and 1 wt% BAPO (photoinitiator for resin photopolymerization) were added to the resin matrix, which was composed of TEGDMA and Bis-GMA at a mass ratio of 2:3. Based on the screening results in Section 2.4, 6 wt% METAC-loaded microcapsules were selected for resin fabrication. These microcapsules were incorporated into the resin matrix at mass fractions of 0%, 5%, 10%, 15%, and 20%, with the resin specimens designated as QM_0_, QM_5_, QM_10_, QM_15_, and QM_20_, respectively. No other inorganic reinforcing filler was added to the resin. Dental light-curing equipment (Woodpecker LED.B, Guilin, China; wavelength range: 420–480 nm; irradiance: 1000–1200 mW/cm^2^) was used to cure the resin specimens prepared in stainless steel molds. Each surface was irradiated for 40 s. The specimen specifications were as follows: resin bars (25 mm × 2 mm × 2 mm, length × width × thickness) and resin discs (10 mm in diameter × 1 mm thickness). Finally, the specimens were sterilized by autoclaving in sealed sterilization pouches (121 °C, 15 min). Before the antibacterial experiments, additional UV irradiation was administered to ensure surface sterility.

### 2.6. Three-Point Bending Test

The resin bars underwent three-point bending tests (QM_0_–QM_20_, *n* = 4 per group) using a universal testing machine (AG-XPLUS10KN, Shimadzu, Kyoto, Japan). The tests were conducted using a support span of 10 mm and a crosshead speed of 1 mm/min. The maximum load at fracture and the equivalent crosshead displacement were recorded. Equation (2) was used to calculate the flexural strength (*σ*), and Equation (3) was used to determine the elastic modulus (*E*), following the protocols of previous microcapsule-based self-healing dental resin studies [12,19].

*σ* = 3*F*_max_*L*/(2*bh*^2^) (2)

*E* = *L*^3^Δ*F*/(4*bh*^3^∆*y*) (3)

where *F_max_*: maximum load (N); *L*: support span (mm); *b*: specimen width (mm); *h*: specimen thickness (mm); Δ*F*: load within the 5 to 10 N range (N); and Δ*y*: crosshead displacement corresponding to Δ*F* (mm).

### 2.7. LIVE/DEAD Bacterial Staining Assay

Resin discs (*n* = 5 per group) were prepared as described in Section 2.5 and divided into four experimental groups as follows: (i) M_10_-S (containing 10 wt% METAC-free microcapsules, surface-scratched), (ii) QM_10_ (untreated), (iii) QM_10_-S (surface-scratched), and (iv) QM_10_-AS (28 d of aging, surface-scratched). Scratches of the same shape and number were made at the same position with a sterile surgical scalpel blade, after aging and immediately before bacterial inoculation. Each specimen surface was inoculated with a 100 μL aliquot of an *S. mutans* suspension (1 × 10^6^ CFU/mL). The specimens were incubated anaerobically for 24 h, followed by gentle rinsing of the residual bacterial suspension with PBS. *S. mutans* cells were stained using the LIVE/DEAD Bacterial Staining Kit. A fluorescence microscope (Olympus IX73, Tokyo, Japan) was used to capture fluorescence images, with the live cells showing green fluorescence and dead cells showing red fluorescence. ImageJ (version 1.54p, Bethesda, MD, USA) software was used for a semiquantitative analysis of the acquired images.

### 2.8. Bacterial Colony Counting

Resin discs (*n* = 3 per group) were prepared and categorized as described in Section 2.7. They underwent anaerobic incubation for 24 h, and the surfaces were gently rinsed with PBS. Each specimen was completely immersed in a centrifuge tube that contained 5 mL of BHI broth. The mixture was vortexed at 2500 rpm for 3 min to elute bacteria that adhered to the surface. The resulting suspension underwent serial dilution, agar plating, and CFU quantification (Section 2.4) to assess the antibacterial activity of the specimens. The antibacterial efficiency of the QM_10_-S was calculated using Equation (1), where the control group was M_10_-S.

### 2.9. Cytotoxicity Assay

Resin discs were divided into four groups as follows: (i) QM_10_ and (ii) M_10_ (*n* = 3 per group), (iii) a positive control, and (iv) a negative control. The QM_10_ and M_10_ specimens were sterilized by autoclaving and UV irradiation, followed by surface scoring with a sterile scalpel. For extract preparation, each specimen was immersed in cell culture medium at a 1:8 (*w*/*v*) ratio and incubated at 37 °C in 5% CO_2_ for 24 h. Subsequently, the extract was used undiluted or after dilution to 2- and 4-fold. HaCaT cells in the logarithmic growth phase were seeded in 96-well plates at a density of 5000 cells per well. After 24 h of culture, the cells were treated with 100 μL of the diluted extracts per well. The negative control group received the cell culture medium, and the positive control group received 5% dimethyl sulfoxide. Blank wells containing only pure culture media and no cells were included. After 0 h and 24 h of incubation (separate replicate wells for each time point), 10 μL of CCK-8 reagent was added to each well. Finally, the cells were incubated at 37 °C for 2 h, and absorbance was measured at 450 nm (OD_450_). Equation (4) was used to compute the relative cell viability.

Cell Viability (%) = (OD_experimental_ − OD_blank_)/(OD_negative_ − OD_blank_) × 100% (4)

### 2.10. Self-Healing Efficiency Evaluation

The resin bars were divided into three groups as follows: (i) QM_0_, (ii) QM_10_, and (iii) M_10_. After light curing, the specimens were stored at room temperature for 24 h. Using a low-speed handpiece under continuous water cooling, V-shaped notches were prepared at the center of each specimen, and the notch tip was sharpened with a razor blade. The final crack depth was 600–700 μm. This procedure was based on previously reported protocols [6]. Specimens were obtained from each group and randomly divided into two equal subgroups (*n* = 3 per subgroup). The unhealed subgroup underwent the three-point bending test immediately, whereas the healed subgroup was incubated at 37 °C for 24 h in a dry environment before testing. Three-point bending tests were conducted as described in Section 2.6, and the maximum load at fracture was recorded. Equation (5) was used to compute the fracture toughness (*K*), and Equation (6) was used to calculate the relative self-healing efficiency (*η*) based on the mean *K* values of both subgroups. The *K* values are used for relative comparison among groups and are not regarded as the critical stress intensity factor. The self-healing efficiency represents the relative increase in fracture toughness of healed over unhealed specimens, rather than the recovery of the original, undamaged resin.

*K* = *F*_max_*L*/(*hb*^3/2^) × *f*(*a/b*) (5)

*η* = (*K*_healed_ − *K*_unhealed_)/*K*_unhealed_ × 100% (6)

where *a*: depth of the crack; *f*(*a/b*): geometric calibration factor, *f*(*a/b*) = 3(a/b)^1/2^[1.99 − (a/b)(1 − a/b)(2.15 − 3.93(a/b) + 2.7(a/b)^2^)]/[2(1 + 2a/b)(1 − a/b)^1.5^] [22]; *K_healed_* and *K*_unhealed_: fracture toughness values of the healed and unhealed subgroups, respectively.

### 2.11. Statistical Analysis

All specimens were independently prepared rather than being technical replicates. Results are presented as the mean ± standard deviation (SD). GraphPad Prism 10.6.0 was used for all statistical analyses. Between-group comparisons were conducted using independent samples *t*-tests. Multiple-group comparisons were conducted using one-way analysis of variance (ANOVA). Two-way ANOVA was used for multifactorial data. All multiple comparisons were conducted using Tukey’s and Šídák’s post hoc tests. Statistical significance was defined as * *p* < 0.05, ** *p* < 0.01, *** *p* < 0.001, **** *p* < 0.0001 (progressive increase); ns suggested no significant difference (*p* ≥ 0.05).

## 3. Results

### 3.1. Microcapsule Characterization

**Figure 2:**
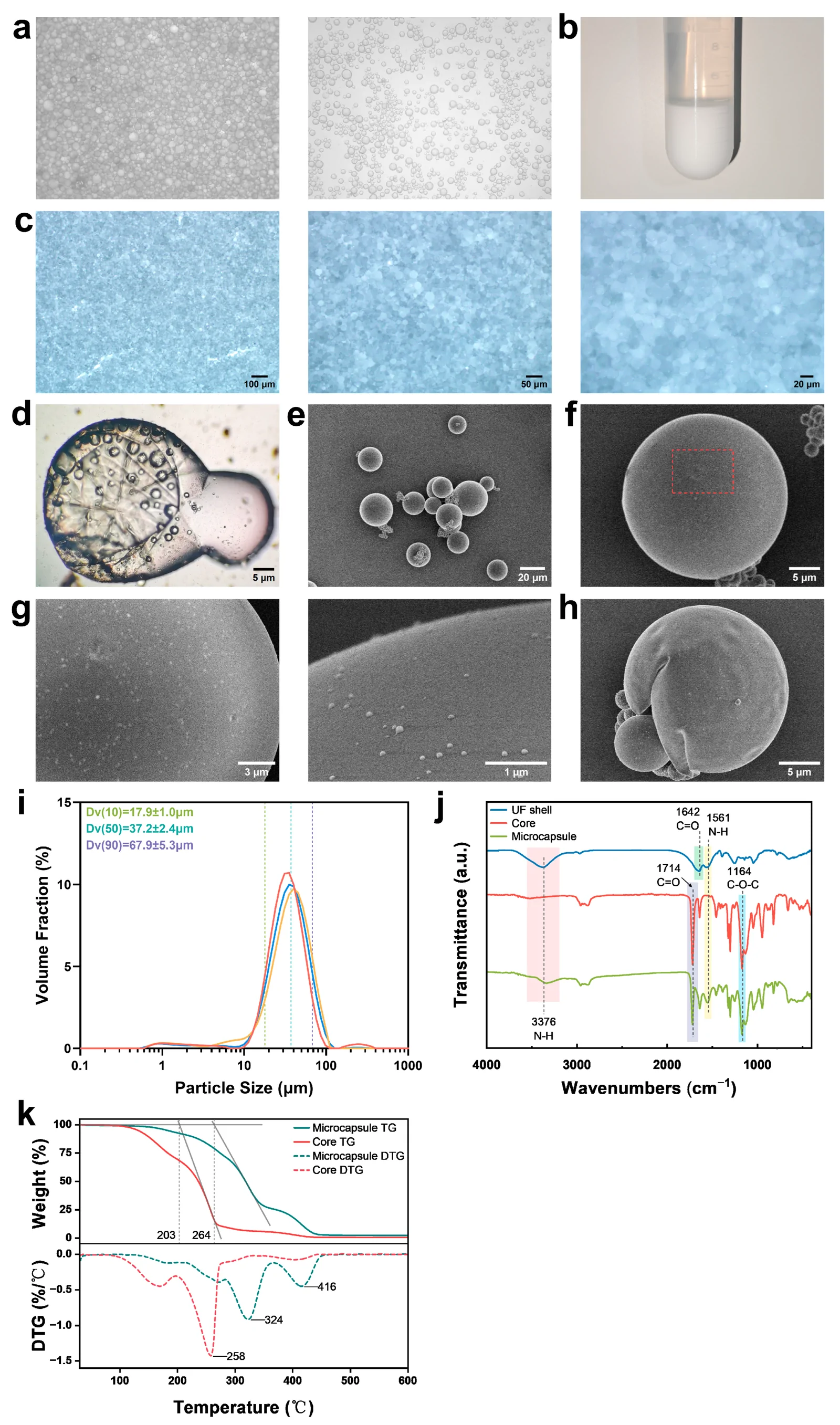
Microcapsule characterization. (**a**) OM images of emulsification and in situ polymerization; (**b**) As-prepared microcapsule samples; (**c**) OM images of dried microcapsules from the same sample at different magnifications; (**d**) OM image of a crushed microcapsule releasing the core material; (**e**–**h**) FE-SEM images of microcapsule morphology and fractured shells; (**i**) Particle size distribution measured by LPSA (colored lines represent three independent measurements); (**j**) FT-IR spectra of the UF shell, core materials, and microcapsules; (**k**) TG and DTG curves of the core material and microcapsules.

Figure 2a illustrates the OM observations acquired during microcapsule synthesis. After emulsification, the core material itself developed stable microspheres without pronounced demulsification. During in situ polymerization, the microcapsules showed favorable dispersion without agglomeration, and the final as-prepared product was visualized as white particles (Figure 2b).

Figure 2c illustrates the OM observations after drying. At low magnification, the microcapsules showed even distribution without noticeable agglomeration. However, at high magnification, the microcapsule shells remained intact without apparent damage. The microcapsules were crushed; the core material was released from the interior, preliminarily suggesting a core–shell structure (Figure 2d).

FE-SEM characterization further confirmed the spherical microcapsule morphology. The shell surfaces were smooth, with limited fine particles attached. Moreover, separate fractures and folds were observed on the shells of ruptured surfaces (Figure 2e–h). The particle-size distribution of the microcapsules was measured by LPSA (*n* = 3) (Figure 2i). The volume-based diameters were as follows: Dv(10) = 17.9 ± 1.0 μm, Dv(50) = 37.2 ± 2.4 μm, and Dv(90) = 67.9 ± 5.3 μm. The Span value was 1.34 ± 0.08, which reflected a narrow particle-size distribution and satisfactory assessment reproducibility.

Figure 2j illustrates the FT-IR characterization results. In the shell material spectrum, a broad N–H stretching vibration peak was observed at 3376 cm^−1^. In contrast, the C=O stretching and N–H bending vibrations of the amide bond yielded absorption peaks at 1642 cm^−1^ and 1561 cm^−1^, respectively. These values are characteristic of UF resin [23]. In the core material spectrum, stretching vibration peaks of ester C=O and C–O–C were observed at 1714 cm^−1^ and 1164 cm^−1^, respectively, which aligned with the TEGDMA and HEMA structures [24]. The complete microcapsule spectrum retained the characteristic peaks of both the shell and core materials, corroborating successful core material encapsulation within the microcapsules.

Figure 2k presents the TGA results. According to the thermogravimetric (TG) curves, the extrapolated onset decomposition temperature of the pure core material was 203 °C, whereas the temperature of the intact microcapsules rose to 264 °C. The mass loss of the microcapsules was approximately 0.9% at 100 °C. The derivative thermogravimetric (DTG) curves demonstrated only one decomposition peak at 258 °C for the core material, whereas two peaks at 324 °C and 416 °C for the microcapsules. Therefore, the UF shell markedly enhanced the thermal stability of the core material.

### 3.2. Antibacterial Activity of Microcapsules

**Figure 3:**
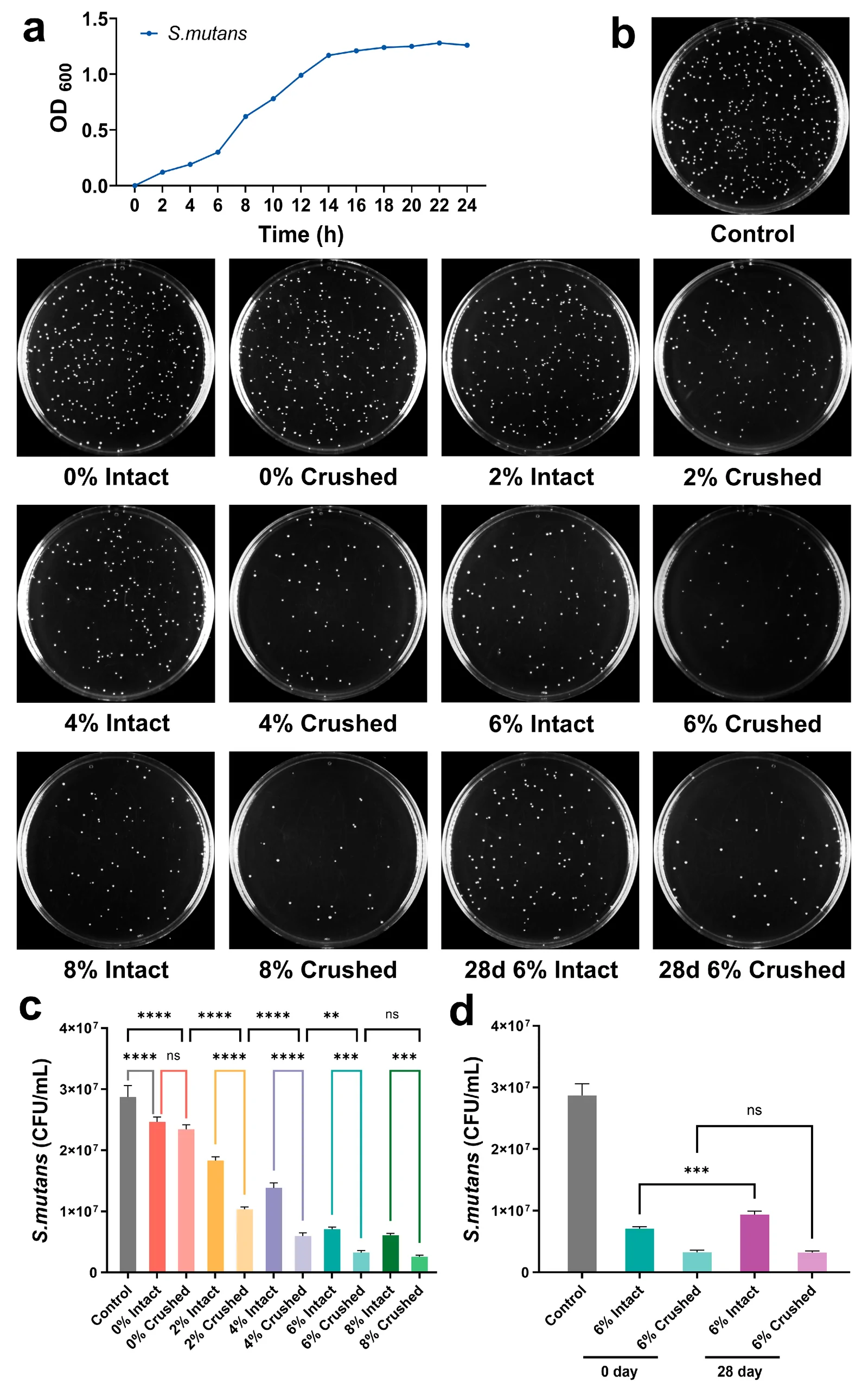
Antibacterial activity of microcapsules. (**a**) *S. mutans* showing 24-h growth curve, monitored by OD_600_; (**b**) Agar culture plates from all experimental groups; (**c**) *S. mutans* colony counts co-cultured with intact and crushed microcapsules at gradient METAC loadings; (**d**) Antibacterial stability of 6 wt% METAC microcapsules before and after 28-day aging. Data are presented as mean ± SD (*n* = 3). Statistical significance was assessed by one-way ANOVA with Tukey’s post hoc test; ** *p* < 0.01, *** *p* < 0.001, **** *p* < 0.0001; ns, not significant.

Figure 3a illustrates the 24-h growth curve of *S. mutans*, which was in line with its typical growth pattern. From 0 to 12 h, *S. mutans* underwent a rapid logarithmic growth phase. After 12 h, the proliferation rate progressively declined, and the culture entered the stationary phase. Figure 3b shows the colony spread plating outcomes for each group. The control group plates showed dense colonies, whereas the experimental groups showed a gradual decrease in colony density with an increase in METAC loading.

Figure 3c shows the quantitative CFU counting results. Compared with the control group, all experimental groups had lower CFU counts (*p* < 0.0001). At the same METAC loading, CFU counts did not significantly differ between the intact and crushed groups at 0 wt% (*p* > 0.05). Nonetheless, the crushed groups exhibited lower CFU counts than the intact groups across the 2 wt% to 8 wt% range (*p* < 0.001), confirming the role of METAC in the microcapsules’ antibacterial activity. Pairwise comparisons of the crushed groups across the METAC-loaded gradient showed significant differences in CFU counts across the 0 wt% to 6 wt% range (*p* < 0.01). However, no significant difference was detected across the 6 wt% to 8 wt% range (*p* > 0.05), with only a marginal increase in antibacterial efficiency observed. The antibacterial efficiencies of the crushed groups for 0 wt%, 2 wt%, 4 wt%, 6 wt%, and 8 wt% loading gradients were 18.45%, 64.04%, 79.23%, 88.75%, and 90.95%, respectively.

Figure 3d shows the antibacterial effects of microcapsules that consisted of 6 wt% METAC after 0 d and 28 d of aging. Compared with 0 d of aging, the intact group demonstrated an increase in the CFU count after 28 d of aging (*p* < 0.001). However, no significant difference was observed in the crushed group (*p* > 0.05), indicating a limitation on premature METAC leakage during aging.

### 3.3. Flexural Properties

**Figure 4:**
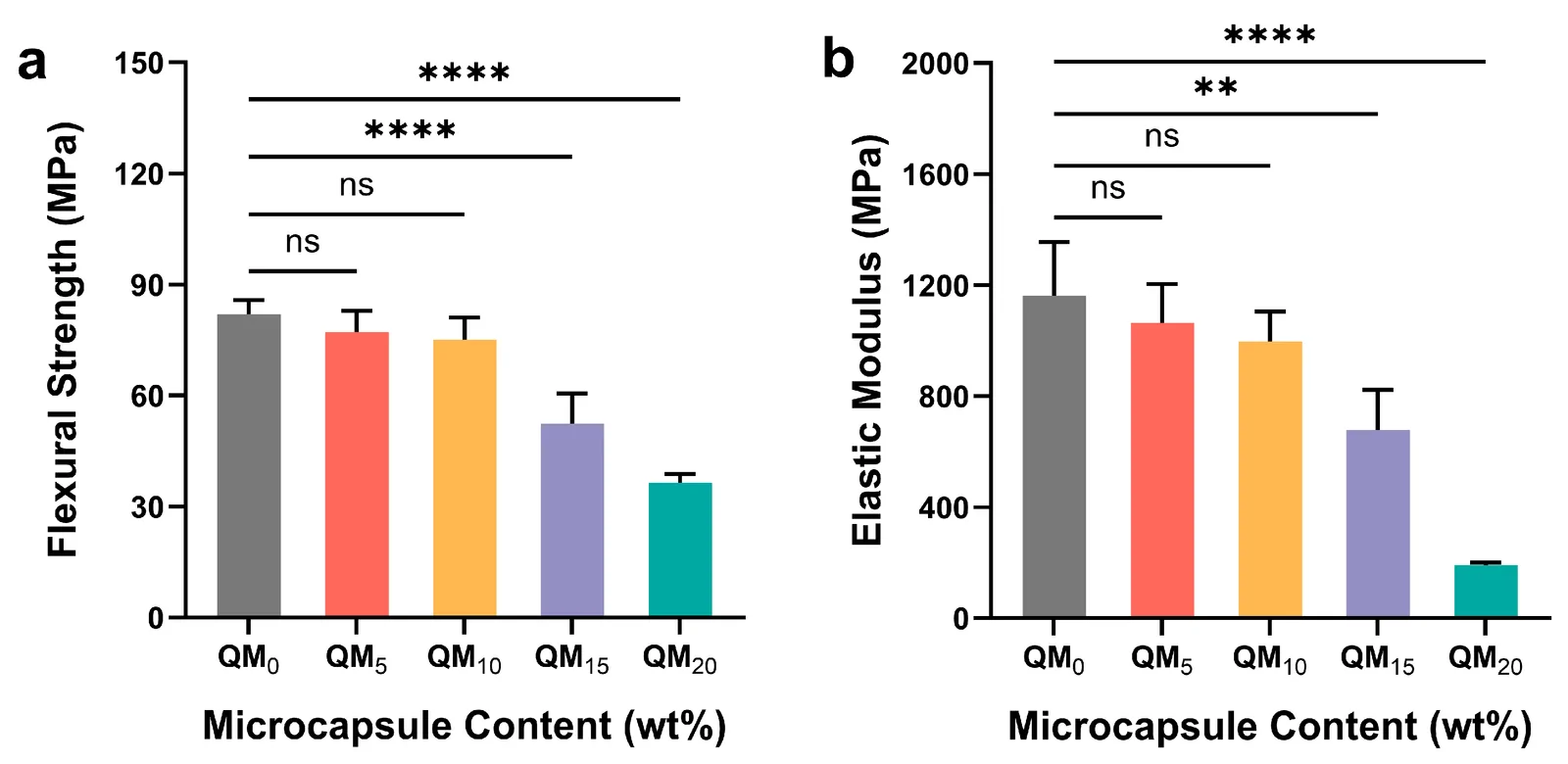
Mechanical properties of the resin specimens. (**a**) Flexural strength at gradient microcapsule loadings; (**b**) Elastic modulus at gradient microcapsule loadings. Data are presented as mean ± SD (*n* = 4). Statistical significance was assessed by one-way ANOVA with Tukey’s post hoc test; ** *p* < 0.01, **** *p* < 0.0001; ns, not significant.

Figure 4 shows the flexural strength and elastic modulus of the specimens. Both mechanical properties decreased gradually with a rise in microcapsule content from 0 wt% to 20 wt%. For flexural strength (Figure 4a), the QM_0_ to QM_20_ groups yielded 82.0 ± 3.8, 77.1 ± 5.8, 75.1 ± 6.0, 52.4 ± 8.1, and 36.4 ± 2.4 MPa, respectively. Elastic modulus demonstrated a comparable trend, with 1162.0 ± 193.3, 1064.0 ± 140.3, 998.1 ± 107.4, 678.2 ± 144.9, and 191.8 ± 8.8 MPa, respectively (Figure 4b). Microcapsule loadings ≤ 10 wt% exerted minimal detrimental effects on mechanical properties, and no statistically significant differences were detected among the QM_0_ to QM_10_ groups (QM_0_ vs. QM_10_: flexural strength *p* = 0.4607, 95% CI: −5.73 to 19.63; elastic modulus *p* = 0.4802, 95% CI: −141.5 to 469.1). In contrast, the QM_0_ group had better flexural properties than the QM_15_ and QM_20_ groups (*p* < 0.01).

### 3.4. Antibacterial Activity of Resin Specimens

**Figure 5:**
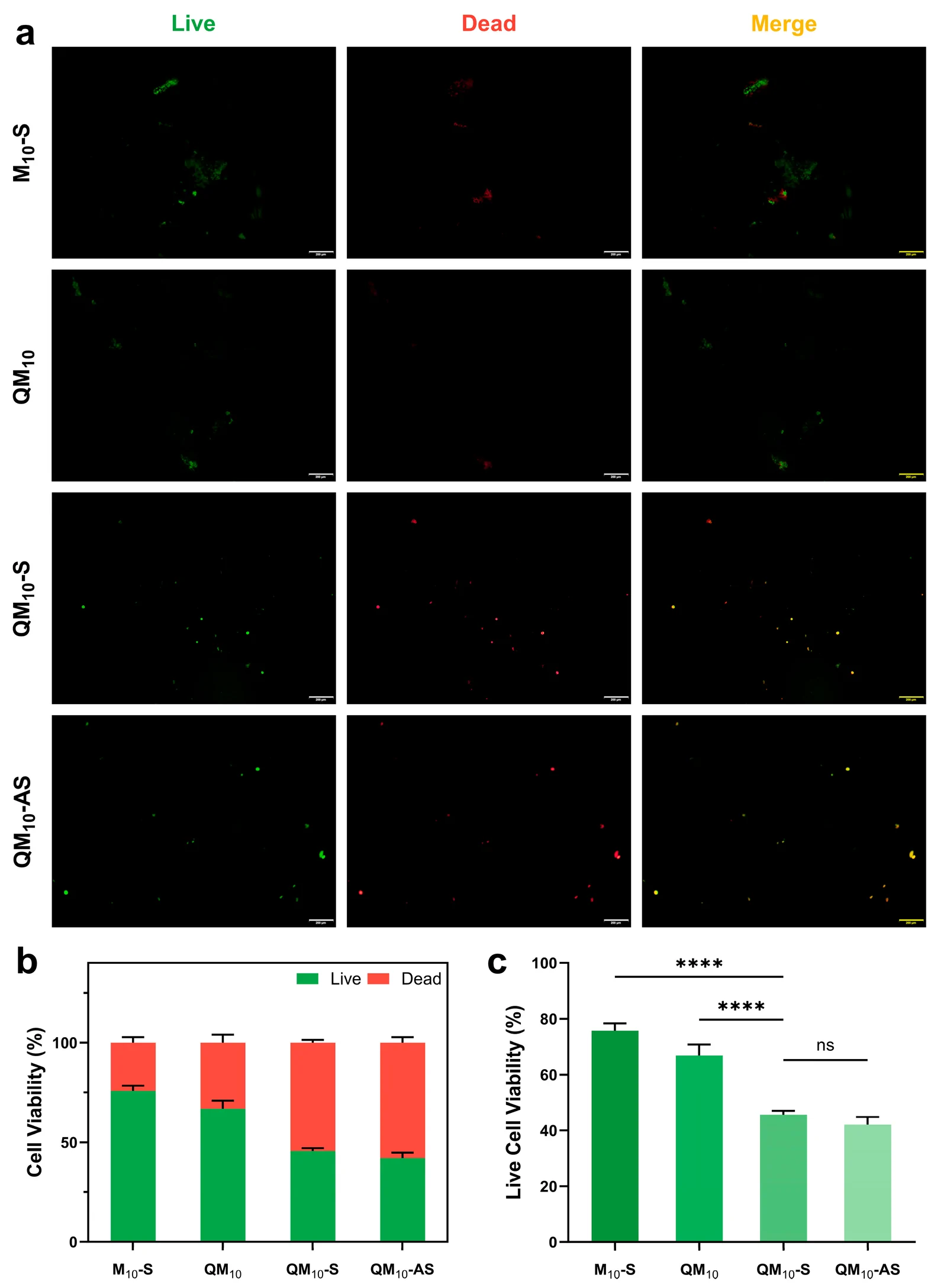
LIVE/DEAD fluorescence staining of resin specimens. (**a**) Fluorescence images of *S. mutans* on specimen surfaces; (**b**) Proportions of live and dead bacterial cells; (**c**) Semi-quantitative analysis of live bacterial viability. Data are presented as mean ± SD (*n* = 5). Statistical significance was assessed by one-way ANOVA with Tukey’s post hoc test; **** *p* < 0.0001; ns, not significant.

Figure 5a illustrates LIVE/DEAD fluorescence staining images of *S. mutans* on the specimen surfaces. The M_10_-S and QM_10_ groups exhibited strong green fluorescence and weak red fluorescence, with the merged images primarily showing green fluorescence. By contrast, the QM_10_-S and QM_10_-AS groups showed reduced green fluorescence and increased red fluorescence, with the merged images showing a marked rise in yellow co-localization signals. According to semiquantitative analysis of bacterial viability (Figure 5b–c), the M_10_-S group had the highest viability, which was markedly reduced in the QM_10_-S group (*p* < 0.0001). Moreover, no significant differences in viability were observed between the QM_10_-S and QM_10_-AS groups (*p* > 0.05).

**Figure 6:**
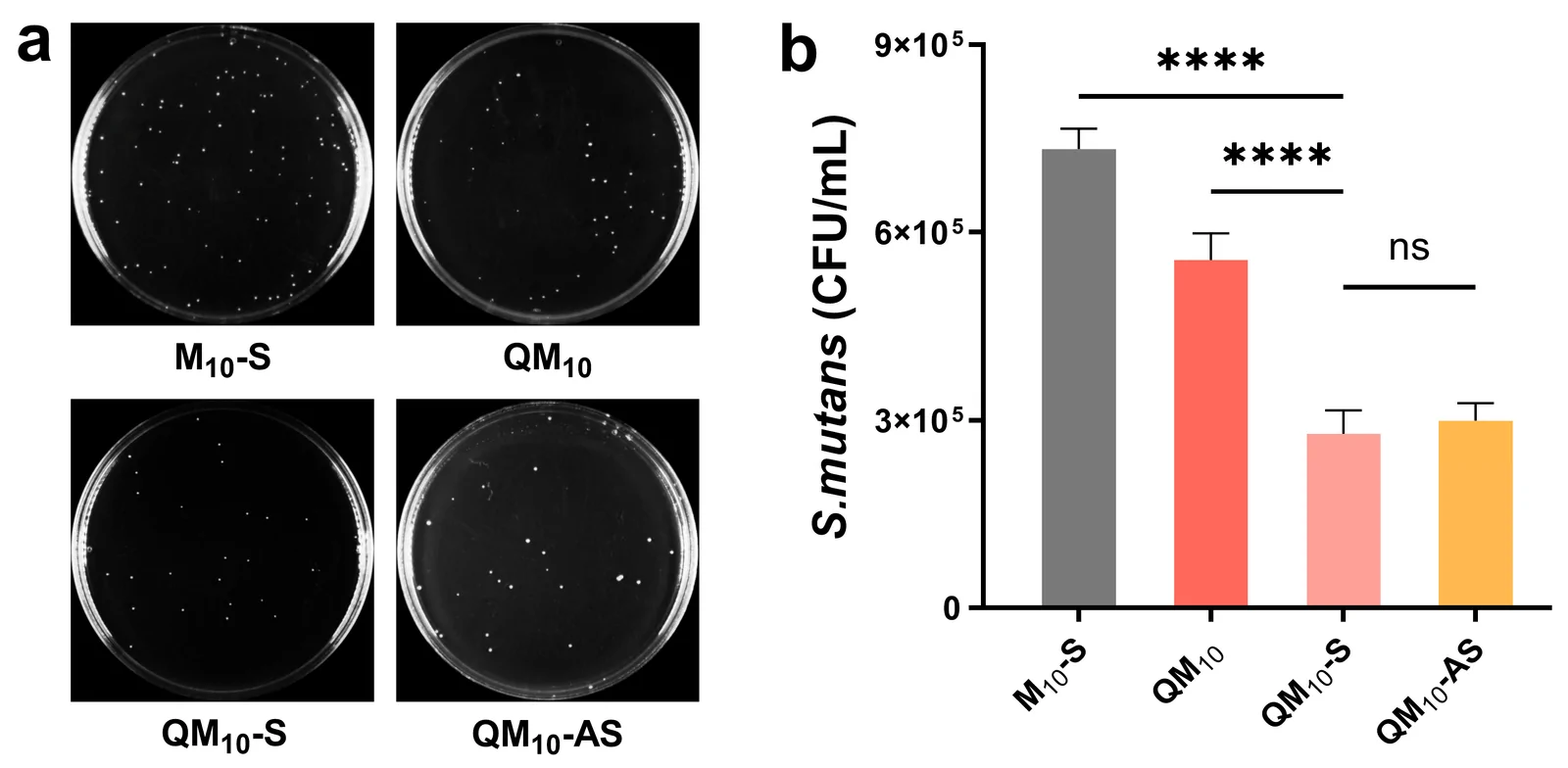
CFU quantification of resin specimens. (**a**) Agar culture plates from all experimental groups; (**b**) *S. mutans* colony counts on specimen surfaces. CFU, Colony-Forming Units. Data are presented as mean ± SD (*n* = 3). Statistical significance was assessed by one-way ANOVA with Tukey’s post hoc test; **** *p* < 0.0001; ns, not significant.

Figure 6a shows the agar plating outcomes of eluted bacteria. The M_10_-S group demonstrated the highest number of colonies, whereas the QM_10_-S and QM_10_-AS groups showed significantly fewer colony numbers. Figure 6b illustrates the CFU count results: the QM_10_-S group had fewer CFU than the M_10_-S group (*p* < 0.0001, 95% CI: 364,024 to 542,643). The antibacterial efficiency of the QM_10_-S group relative to the M_10_-S control was 61.82%. The QM_10_-S group also had fewer CFU than the QM_10_ group (*p* < 0.0001). No significant difference was observed between the QM_10_-S and QM_10_-AS groups (*p* > 0.05). The CFU results and fluorescence staining results were comparable.

### 3.5. Cytotoxicity of Resin Specimens

**Figure 7:**
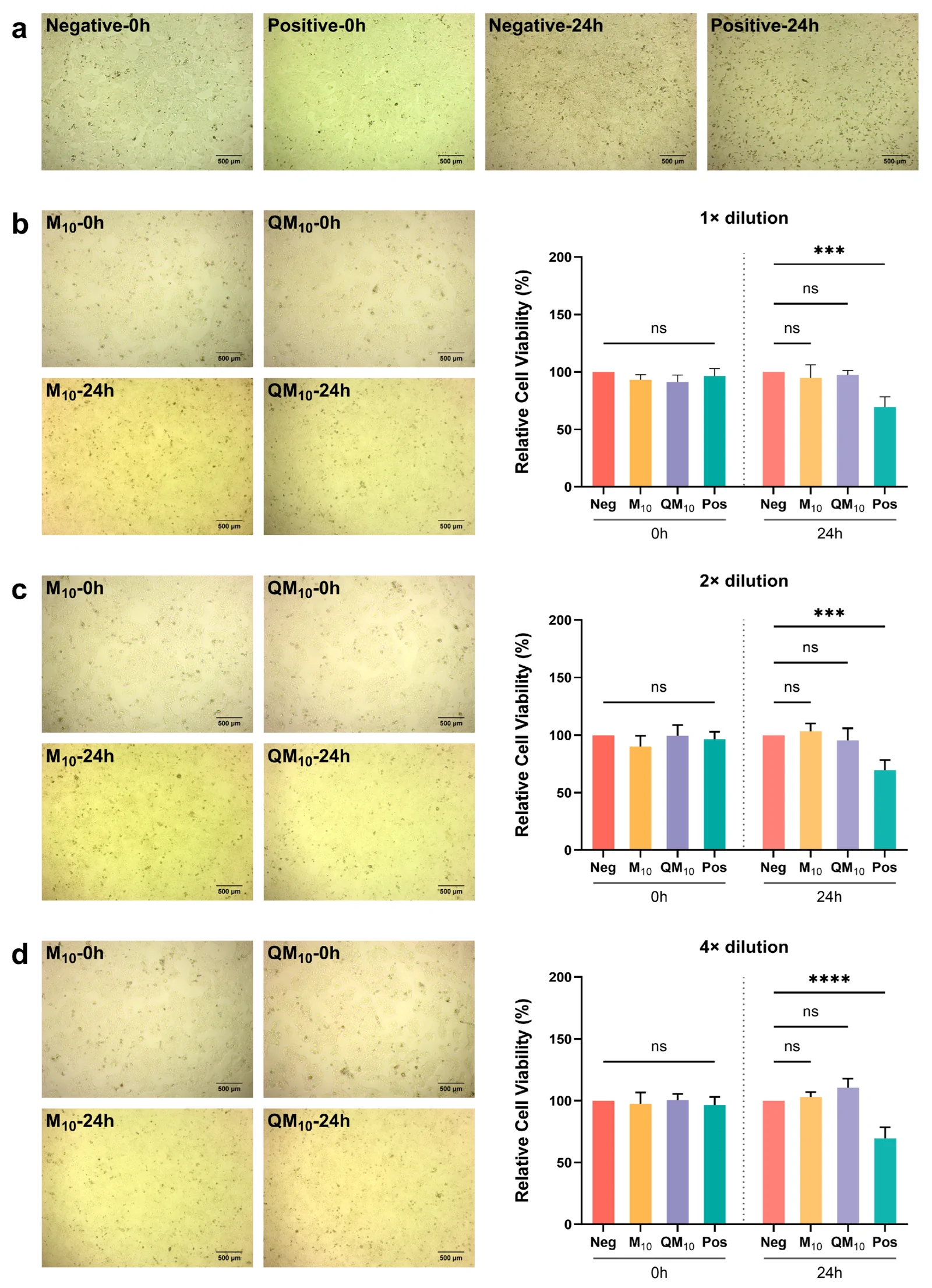
Cytotoxicity of resin specimens. (**a**) HaCaT cells in both negative and positive control groups at 0 h and 24 h; (**b**–**d**) Images and relative viability of HaCaT cells in all experimental groups at dilution ratios of (**b**) 1×, (**c**) 2×, (**d**) 4×. Data are presented as mean ± SD (*n* = 3). Statistical significance was assessed by two-way ANOVA with Tukey’s post hoc test; *** *p* < 0.001, **** *p* < 0.0001; ns, not significant.

Figure 7a illustrates the morphological findings of HaCaT cells in both positive and negative control groups. Cells in both groups showed well-spread morphology with strong substrate attachment at 0 h. At 24 h, cells in the negative control group remained intact and adherent. In contrast, cells in the positive control group appeared shrunken and rounded, with a marked increase in cell detachment and cellular debris.

Figure 7b–d displays the cell morphology and relative viability outcomes for the M_10_ and QM_10_ groups, which correspond to total dilution factors of 1, 2, and 4, respectively. The M_10_ and QM_10_ groups showed uniformly dispersed cells with typical morphology at 0 h. At 24 h, both groups showed adherent cells and formed a dense monolayer, without any discernible morphological differences between the groups. Quantitative OD_450_ measurements demonstrated consistent trends across all dilution levels. At 0 h, no difference was observed in relative cell viability among all groups (*p* > 0.05). At 24 h, no significant difference was detected among the M_10_, QM_10_, and negative control groups (*p* > 0.05). The positive control group exhibited lower cell viability than the remaining groups (*p* < 0.001).

### 3.6. Self-Healing Efficiency of Resin

**Figure 8:**
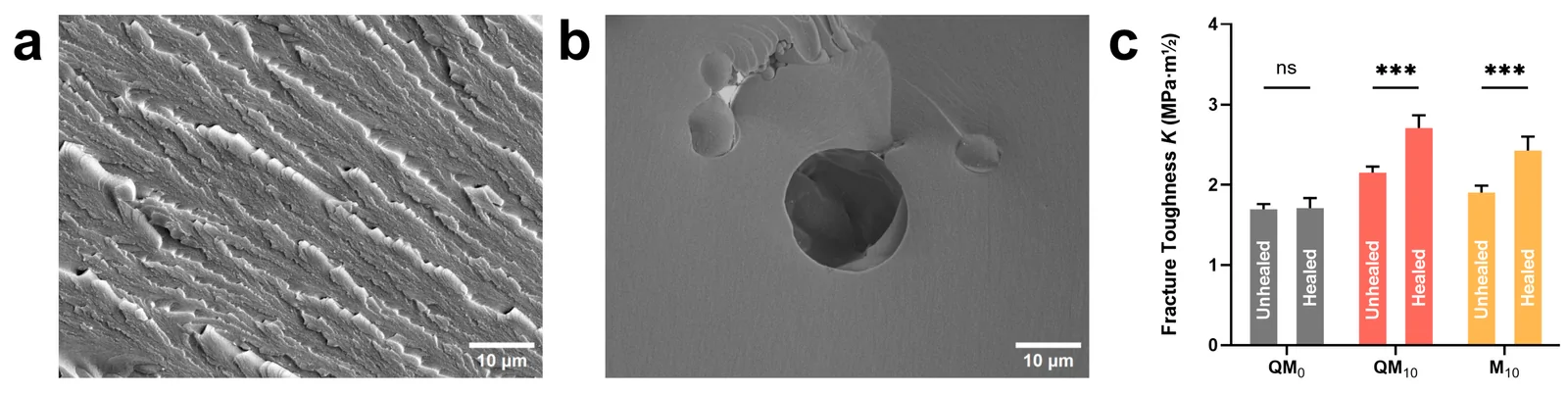
Self-healing performance of the resin specimens. (**a**) FE-SEM image of fracture surfaces from the QM_0_ group; (**b**) FE-SEM image of fracture surfaces from the QM_10_ group; (**c**) Fracture toughness of healed vs. unhealed specimens for different resin groups. Data are presented as mean ± SD (*n* = 3). Statistical significance was assessed by two-way ANOVA with Šídák’s multiple comparisons test; *** *p* < 0.001; ns, not significant.

Figure 8a,b shows FE-SEM micrographs of the specimen fracture surfaces. The QM_0_ group demonstrated several river-like fracture steps, which typically develop during crack propagation. However, microcapsule structures were absent (Figure 8a). In the QM_10_ group, fracture surfaces showed circular pits with distinct borders, which were typical of structures retained after microcapsule rupture. The river-like fracture steps extended to the pit margins without crossing the pit regions (Figure 8b).

Figure 8c illustrates the fracture toughness of the specimens. For QM_10_, the healed subgroup showed significantly higher fracture toughness than the unhealed subgroup (2.71 ± 0.16 vs. 2.15 ± 0.08 MPa·m^1/2^; *p* = 0.0004, 95% CI: 0.28 to 0.83). Similarly, for M_10_, the healed subgroup showed significantly higher fracture toughness than the unhealed subgroup (2.43 ± 0.17 vs. 1.90 ± 0.09 MPa·m^1/2^; *p* = 0.0006, 95% CI: 0.25 to 0.80). However, no significant difference was found between the healed and unhealed subgroups of the QM_0_ group (1.71 ± 0.13 vs. 1.70 ± 0.06 MPa·m^1/2^; *p* = 0.9988, 95% CI: −0.26 to 0.29). Based on the fracture toughness results, the self-healing efficiencies of QM_10_ and M_10_ were 25.7% and 27.4%, respectively, whereas QM_0_ showed nearly 0% (0.8%). Moreover, the increase in fracture toughness after healing did not differ significantly between QM_10_ and M_10_ (interaction *p* = 0.8385).

## 4. Discussion

In this study, TEGDMA, HEMA, and METAC were used as the core materials in an in situ polymerization process to create microcapsules with a UF resin shell. An experimental dental resin with both self-healing and antibacterial properties was fabricated by incorporating these microcapsules into a Bis-GMA/TEGDMA resin matrix. The particle size and dispersion of microcapsules directly affect the mechanical properties of dental resins. These properties can be compromised by stress concentrations caused by disproportionately large particle size and poor dispersion [22]. The prepared microcapsules showed a narrow particle size distribution and good dispersion, which facilitated maintaining the mechanical stability of the dental resin. Furthermore, the FT-IR spectra were compared between the core materials and the microcapsules; the UF shell encapsulated the core materials. Meanwhile, all of the core material’s characteristic absorption peaks remained visible in the microcapsule spectra. No new distinctive peaks were observed, indicating that the core-related functional groups were preserved. The ability of the microcapsules to conduct their intended self-healing and antibacterial capabilities depends on the intact functional groups. According to TGA results, the microcapsules exhibited a two-step thermal decomposition profile, which aligned with findings for comparable microcapsule systems [7]. UF shells significantly improved the thermal stability of the core materials. Therefore, the microcapsules might withstand the light-curing of the dental resin, thus preventing premature leakage and core material degradation.

MDPB [25], K18 [26], DMADDM [27], and DHMAI [28] are among the QAMs that have been developed for use in dental restorative materials. These components comprise polymerizable double bonds and quaternary ammonium groups. They can crosslink and copolymerize with acrylate monomers to render the resin with long-term antibacterial properties [29]. METAC, a monofunctional polymerizable quaternary ammonium monomer, consists of one methacryloxy group and one quaternary ammonium cationic group. Garcia confirmed that METAC can copolymerize with both Bis-GMA and TEGDMA. It can alleviate stress concentrations attributed to poor component compatibility while having no discernible impact on the resin’s double-bond conversion [30]. According to Kohno, polymerized METAC exerted a strong bactericidal effect on *S. mutans*, decreasing the colony count by 2.83 log units [31]. In our study, gradient loading assays demonstrated that the microcapsules’ antibacterial activity increased gradually with the METAC loading and plateaued at 6 wt%. Further increases in loading did not significantly improve the antibacterial effect. Given the monofunctional nature of METAC, higher METAC loading may compromise the mechanical performance of the polymer network [13]. Consequently, 6 wt% was deemed a suitable loading ratio within the investigated range. Compared with intact microcapsules, crushed ones exhibited significantly higher antibacterial activity at the same loading level. Antibacterial activity did not significantly differ between intact and crushed microcapsules in groups without METAC loading, indicating that the enhanced antibacterial activity of the crushed microcapsules was attributable to METAC release. According to aging tests, the elution of surface-adsorbed free METAC may have contributed to the marginal decline in the antibacterial activity of intact microcapsules. In contrast, microcapsules that ruptured after aging showed no noticeable decline in antibacterial activity, indicating minimal leakage of the core material. This is consistent with the encapsulation of METAC within the UF shell. The microcapsule carrier system is widely applicable to a variety of antibacterial agents. The maximum loading of antibacterial agents can be further increased through the development of QAMs with low viscosity and high double bond conversion, improving the dental resin’s antibacterial efficacy.

The incorporation of microcapsules into a resin matrix affects the mechanical properties of the dental resin [32]. To identify a suitable loading ratio, we established groups with varying loading gradients. Both the flexural strength and elastic modulus of the resin decreased gradually with an increase in microcapsule loading. Both mechanical indicators markedly declined with a >10 wt% increase in the loading content, which is in line with other research findings [7,33,34]. This decline was probably caused by greater particle agglomeration at higher microcapsule loadings, which raised the risk of resin fracture, disrupting the matrix’s structural continuity. Additionally, factors, including capsule wall stiffness, particle size, dispersion uniformity, and strength of the filler-resin interfacial bond, can directly limit the optimal microcapsule loading [8,10,35]. Overall, 10 wt% was deemed a practical loading content given the conditions of this study, considering the changes in mechanical properties. Nevertheless, the lack of a statistically significant difference between the QM_0_ and QM_10_ groups does not necessarily indicate mechanical equivalence, as microcapsule incorporation may affect matrix continuity and local stress distribution [36].

Self-healing efficiency is a key metric for evaluating the self-healing performance of dental resins. However, the testing strategy and evaluation models can substantially affect the measured efficacy, leading to variations across studies [32]. The *η* value obtained in this study represents an experimental measure under the specific notch and healing protocol employed, rather than an intrinsic restorative performance. Uniform V-notches were inserted into parallel specimens to mirror the early-stage microdamage in clinical restorations and simulate self-healing. The unchanged fracture toughness of QM_0_ after incubation ruled out continued curing or storage effects. The resin containing 10 wt% microcapsules therefore exhibited self-healing capacity. Moreover, no significant difference was observed between the QM_10_ and M_10_ groups in the fracture toughness gain after healing, indicating that METAC did not adversely affect self-healing. A possible explanation is the presence of a methacrylate group in METAC. It can be incorporated into methacrylate-based monomer blends and does not affect the polymerization process [31,37]. Furthermore, the low METAC loading caused minimal disruption to the cross-linked network. Cross-sectional observation showed that microcrack propagation was related to microcapsule rupture. This damage-triggered microcapsule rupture may provide a basis for potential crack healing of the dental resin.

The antibacterial functionalization of microcapsules is an effective approach to prevent secondary caries. Yao inhibited *S. mutans* by grafting nano-antibacterial materials (NIFs) onto microcapsule surfaces [19]. Tung oil’s inherent antibacterial activity and oxidative polymerization qualities have been used in other studies to encapsulate the oil within microcapsules and accomplish both self-healing and antibacterial properties [38]. In our study, METAC-loaded microcapsules were fabricated to establish a microcrack-triggered antibacterial system. The antibacterial agent is sealed within the microcapsules when the resin structure is intact and is released upon microcapsule rupture induced by microcrack propagation. Together, the fluorescence staining and colony counting results showed that the QM_10_-S group exhibited higher antibacterial activity than the QM_10_ group, supporting the feasibility of the proposed method, and this method is expected to enhance the antibacterial activity of the dental resin after damage. However, as scratching may also alter surface roughness, exposed surface area, and bacterial adhesion, the difference cannot be attributed solely to METAC release.

The clinical translation of dental materials necessitates biosafety. Here, HaCaT cells were co-incubated with resin specimen extracts at different dilution ratios. Consistent with earlier findings [10,19], cells in the experimental group exhibited favorable adhesion and proliferative activity, with no significant difference in relative cell viability compared with the negative control group. Thus, the dental resin containing the microcapsules showed no discernible cytotoxicity in the tested diluted extracts, HaCaT cells, and 24-h exposure duration. These results could be explained by two factors. First, the core material may not be released widely in intact microcapsules. Second, METAC may show low cytotoxicity owing to its short alkyl chain quaternary ammonium salt structure. Garcia confirmed that low METAC concentrations did not cause significant cytotoxicity in HaCaT cells [30].

Despite the promising results obtained in the present study, several limitations should be acknowledged. Regarding the resin formulation, the experimental resin contained only the resin matrix and microcapsules without conventional inorganic fillers, and the present mechanical results therefore cannot be directly extrapolated to commercial restorative composites. In addition, direct quantitative analysis of METAC encapsulation efficiency, release, and degree of conversion was not performed, which limits our full understanding of the self-healing and antibacterial mechanisms; given that the release behavior of resin-based restorative materials depends on the material and time [39], such analyses are valuable and should be included in future studies. As for the antibacterial evaluation, the activity was assessed against a single cariogenic species (*S. mutans*). Furthermore, the present study did not include factors relevant to secondary caries in the oral environment, such as multi-species biofilms, salivary pellicle, dynamic nutrient exposure, pH cycling, mechanical wear, or repeated bacterial challenges. Evaluation under these conditions would provide stronger evidence for the clinical relevance of the present findings. Moreover, the aging period was limited to 28 days in our study. A recent long-term study showed that the release behavior of resin-based restorative materials varies with both the aging time and the material [40]. Therefore, the long-term stability of the antibacterial activity remains to be verified. The self-healing process was assessed under dry conditions with a single damage event, and its actual efficacy under complex oral conditions and over frequent damage cycles remains to be explored. Finally, the cytotoxicity assessment was limited to a 24-h exposure with a single cell line, and further evaluation with additional cell lines and longer exposure periods is warranted.

## 5. Conclusions

In summary, dual-functional microcapsules were fabricated by encapsulating METAC/TEGDMA/HEMA within UF resin shells. The microcapsules showed a regular morphology, uniform particle size distribution, and high thermal stability. Under the present experimental conditions, the 6 wt% METAC loading and the 10 wt% microcapsule incorporation ratio provided the most favorable balance among the mechanical, antibacterial, and self-healing outcomes evaluated. The resultant experimental dental resin demonstrated promising self-healing efficiency and antibacterial properties, along with no discernible cytotoxicity. As the experimental dental resin contained only the resin matrix and microcapsules, the present results cannot be directly extrapolated to commercial restorative composites. The findings provide proof-of-concept evidence that METAC-loaded microcapsules can combine self-healing behavior with enhanced antibacterial activity after mechanical disruption in an experimental methacrylate resin. Further studies incorporating direct release measurements, polymerization analysis, longer and more clinically representative aging, cyclic fatigue, multi-species biofilms, and broader biocompatibility testing are required before the clinical potential of this system can be established.

## Data Availability

The raw data supporting the conclusions of this article will be made available by the authors on request.

## Abbreviations

The following abbreviations are used in this manuscript:

CFU	Colony-Forming Units
METAC	[2-(Methacryloyloxy)ethyl]trimethylammonium chloride
OM	Optical Microscopy
FT-IR	Fourier Transform Infrared
FE-SEM	Field Emission Scanning Electron Microscopy
TG	Thermogravimetric
DTG	Derivative Thermogravimetric
UF	Urea–Formaldehyde
LPSA	Laser Particle Size Analysis
